# Supplementary material for: Skeletal development in the sea urchin relies upon protein families that contain intrinsic disorder, aggregation-prone, and conserved globular interactive domains
Source: PLoS One. 2019 Oct 1;14(10):e0222068. doi: 10.1371/journal.pone.0222068 (PMC6771980; doi:10.1371/journal.pone.0222068)
Supplement: Attachment — Submitted filename: Response.pdf [file pone.0222068.s001.pdf]

Response to reviewers

Reviewer 2

1 The reviewer states, "*In addition to far-UV CD and 1H-NMR analyses, structure of SpSM50 and SpSM30B/C (S. purpuratus) should be probed by near-UV CD spectroscopy (320-250 nm). This will provide important information on the environment of aromatic residues in the gel state of these proteins*."

Response: This is a good idea, and in fact in previous papers we have addressed aromatic residues (see *Biochemistry* 2014, **53**, 2739-2748; *Biomacromolecules* 2014, **15**, 4467-4479; *ACS Omega* 2017, ***2***, 6151-6158). However, as stated in the introduction (see pp 3,4) our present focus is to address the structural features that exist amongst SM proteins, and, elucidate the basis for spicule matrix protein hydrogel-water binding and release. Moreover, aromatic CD experiments would be qualitative in nature, whereas the NMR experiments that we can employ are a bit more quantitative and thus the Asn, Gln, Arg study is more feasible at the moment. We do intend to study aromatic residues using NMR at a later time.

2 The reviewer states, "*I would recommend to analyze secondary and tertiary structures of SpSM50 and SpSM30B/C (S. purpuratus) in their non-gel states (i.e., under the conditions that do not promote hydrogel particle formation). This can be done using far-UV and near-UV CD spectroscopy, respectively. This analysis will provide an important information on the effect of gelation on structural properties of these proteins*."

Response: This is a good idea however, these proteins are very strong aggregators in all buffers and so far we have not been able to identify conditions where a monomeric form of SpSM50 or SpSM30B/C exists, so unfortunately we cannot perform these studies at this time.

3 The reviewer states, "*The authors are encouraged to utilized CH-CDF plot method (PMID: 22174269) in analysis of nine spicule matrix proteins listed in Table 1. This will provide an important information on the sub-classification of the overall disorder status of these proteins*."

Response: This is an excellent suggestion and we have now generated a CH-CDF plot (new Figure 5) and discussed its relevance for the spicule matrix proteome (see materials and methods, pp 6-7; results with Figure 5, pg 11; and brief discussion on pg 18).
